# Molecular Mechanism of Muscle Contraction: New Perspectives and Ideas

**DOI:** 10.1155/2015/694345

**Published:** 2015-04-19

**Authors:** Oleg S. Matusovsky, Olga Mayans, Danuta Szczesna-Cordary

**Affiliations:** ^1^Meakins-Christie Laboratories, McGill University, Research Institute of the MUHC, Centre for Translational Biology, Montreal, QC, Canada H4A 3J1; ^2^A.V. Zhirmunsky Institute of Marine Biology, FEB RAS, Vladivostok 690041, Russia; ^3^Institute of Integrative Biology, University of Liverpool, Liverpool L69 7ZB, UK; ^4^Department of Molecular & Cellular Pharmacology, University of Miami Miller School of Medicine, Miami, FL 33136, USA

Movement, motility, and conformational dynamics of proteins are the basic phenomena of life underlying various physiological and pathological processes of the cell. Muscle cells use an extensively organized but still poorly understood actin-myosin contractile apparatus to generate force and movement and respond to various physiological stimuli. In this special issue we present several invited original and review articles from researchers who study muscle structure and function from different perspectives and emphases.

During the last decades we have seen a major revision of the immutable postulates underlying the molecular mechanisms of muscle contraction. One of the best examples is the discovery of giant proteins widely distributed in nature from nematodes to humans. It was shown that giant proteins are involved in some* fascinating phenomena*; molluscan twitchin regulates the so-called catch state, which permits generation of force with little expenditure of energy. The twitchin-like protein in insects called projectin is implicated in stretch activation of flight muscle, which allows insects to beat their wings at frequencies higher than possible by neural stimulation. The function of titin, the biggest protein involved in various processes in vertebrate skeletal muscle including sarcomere assembly, passive elasticity, myofibrillogenesis, gene regulation, and signaling in health and disease, is still not fully understood.

J. Bogomolovas et al. evaluated the clinical potential of titin ligands in the progression of cardiac remodeling associated with the end-stage idiopathic dilated cardiomyopathy (IDCM). The authors showed that one of the tested ligands, ANKRD1, is a potential marker for cardiac remodeling and disease progression in IDCM and that its greater expression correlated with reduced cardiac contractility.

In skeletal muscle, titin spans each half-sarcomere from the Z-band to M-line. The review of L. Y. Hu et al. is an elegant summary of the recent vast volume of data concerning the M-band region of striated muscle. While the role of the M-region in supporting myofibrillar structure and contractility is well established, its role in mediating additional cellular processes has only recently started to emerge. As such, the M-region is a hub of key protein players contributing to cytoskeletal remodeling, signal transduction, mechanosensing, metabolism, and proteasomal degradation.

Another comprehensive review by A. Månsson et al. pointed out the poorly understood aspects of striated muscle contraction and, in particular, the relationship between the force generation process and the phosphate-release step in the ATP-turnover cycle. The authors discuss in-depth the role of the two heads of myosin II and whether they are independent or cooperative in their interaction with actin. The structure and function of actin filaments in muscle contraction are also discussed.

Four original research articles in the issue were devoted to heart muscle and cardiac disease. J. Liang et al. tested the effect of the hypertrophic cardiomyopathy-linked mutation in human cardiac troponin I (cTnI) on the contractile properties and myofilament protein phosphorylation in papillary muscle preparations from the left (LV) and right (RV) ventricles of R21C+/+-knock-in mice. The authors showed a significant functional difference in contractile force generation between the LV and RV in the disease model which is not present in wild-type mice. They concluded that the presence of the effect of the mutation only in the LV is a testimony to the power of the mutation exerting its detrimental effects on the function of the LV.

H. B. Lin et al., in their original study, showed that inhibition of MMP-2, the matrix metalloproteinase, is instrumental in enhancing cardiomyocyte contractility and protecting the heart against simulated ischemia-reperfusion (I/R) injury. In another paper by D. Bialy et al., the authors tested the effects of low frequency electromagnetic field conditioning in a model of ex vivo cardiac I/R and demonstrated that when applied prior to, during, and after the ischemic insult, it protects the heart against I/R-induced cardiac contractile dysfunction.

In a well-executed work, K. M. Haizlip et al. showed that during muscle contraction force and calcium transients are not changing in parallel and that a change in steady-state conditions occurs in multiple phases. A rapid phase, characterized by a fast change in force production, is mirrored by a change in calcium transient amplitude. But during a slow phase that occurs as the muscle proceeds to stabilize at the new frequency, dissociation between the calcium transient amplitude and developed force occurs. This dynamic relationship between force and calcium upon a switch in stimulation frequency unveils the dynamic involvement of a myofilament-based regulation during a change in the cardiac contractile steady-state.

The paper of J. Karolczak et al. addressed a unique actin-based motor protein, myosin VI, and performed a search for its binding partners in mouse myoblasts and myotubes. A kinase anchoring protein 9 (AKAP9), a regulator of the protein kinase A (PKA) activity, was identified as a binding partner for myosin VI. The authors concluded that this novel interaction between myosin VI and AKAP9 links myosin VI with the PKA pathway, which could be important for the regulatory activity of myosin VI in the cell.

The present issue constitutes an important update in a constantly developing field. The efforts to carry on these studies may generate new opportunities in the near future.

